# Hospital Meetings, &c.

**Published:** 1900-02-03

**Authors:** 


### The Institutional Workshop.

### HOSPITAL MEETINGS, &C.

### GERMAN HOSPITAL.

The annual meeting of the German Hospital was held on Friday last, 26th ult., at Winchester House, E.C., Baron Von Schröder, the treasurer, presiding.

The committee, in the report presented, referred with great regret to the loss of several valued members of the working staff, among them Dr. Port, for over thirty years one of the medical officers, and lately senior hon. physician; the Rev. C. Schoell, upwards of forty years an active, and lately the oldest, member of the committee; Sir James Paget, the hon. consulting surgeon; and their venerable and much-respected matron, Miss Christiane Bürger, who for upwards of fifty-nine years conscientiously attended to her duties in the hospital and convalescent home. The report also stated that Mr. Edward A. Grüning, the hon. architect and surveyor for thirty-five years, had resigned, Mr. C. G. F. Rees, of the Estate Office, Stanmore, being apppointed to the post. Among other changes recorded were the following: Mr. R. W. Parker, senior honorary surgeon, having resigned at the beginning of the year, has been elected honorary consulting surgeon to the hospital. Through the death of Dr. Port and the resignation of Mr. Parker, Dr. F. Parkes Weber has become senior honorary physician, and Dr. E. Michels senior honorary surgeon. The resident medical officers, Dr. R. Wunsch, Dr. W. Hirt, and Dr. O. Müller, having resigned and returned to Germany, their places have been filled by Dr. Wilhelm Scheffer, from Pfungstadt, near Frankfort, and Dr. Felix Kraemer, from Berlin, as resident medical officers, and by Dr. Franz Dengler, from Landau, as assistant resident medical officer. The head sister, Miss Johanna Schürmann, has been compelled, through family reasons, to resign the post which she has so well filled for the last five years, and Sister Ida Mohn has been appointed head sister in her stead. The committee also proposed that Dr. Karl Fürth, hitherto honorary assistant physician, be elected honorary physician; Dr. Rudolf Gruber, M.R.C.S.Eng., L.R.C.P.Lond., of 67, Wimpole Street, W., honorary ophthalmic surgeon; and Dr. J. L. Bunch, M.R.C.P.Lond., of 32, Wimpole Street, W., honorary assistant physician to the hospital. As regards the working of the hospital, the committee reported that during the year there had been admitted 1,697 in-patients—of which 206 were cases of accident, nearly all English—as compared with 1,913 in 1898. Of these 78 were sanatorium patients, and 499, after leaving the hospital, were admitted into the convalescent home. The out-patients —the greater part English—numbered 24,549, making altogether 26,246 patients treated during the year, as against 27,635 in 1898. In response to the request made by representatives of a committee formed for the establishment of a Kosher kitchen for Jewish patients in the German Hospital, the committee announced that they had accepted the proposal, on condition that the hospital should be indemnified for all expenses incurred for additional buildings, fittings, and maintenance. The required sums having been promised, the establishment of the kitchen will soon be an accomplished fact. The committee also desired to put on record their thanks to Mr. Alexander Siemens, one of their colleagues, for the gift of a complete Röntgen rays apparatus, which was certain to prove a great acquisition to the hospital. The finances of the hospital were, the committee reported, on the whole, satisfactory. The loan of £250 from the bankers in 1898 had been repaid, but they regretted having been compelled to use the full amount of legacies—£1,508—received during the year to meet current expenses, instead of investing them as they would have liked to do. The amount collected at the annual dinner on April 17th was £3,035. Annual subscriptions totalled £1,445, as compared with £1,488 in 1898, a decrease partly accounted for by the Manchester subscriptions not having been remitted until January, too late for inclusion. The committee acknowledged the receipt of £625 from the Metropolitan Hospital Sunday Fund and £164 from the Hospital Saturday Fund. The receipts from the sanatorium were £513, as compared with £524 in the previous year. The ordinary income, including the £1,508 from legacies, totalled £9,911. The ordinary expenditure was £9,587; the extraordinary (building improvements) £102.

The Chairman very briefly moved the adoption of the report and financial statement, and this was carried. The election of the Rev. G. Maetzold and Mr. F. A. Konig as members of the committee was confirmed; and votes of thanks to the staff and to the chairman closed the proceedings.

---

### ITALIAN HOSPITAL.

The annual general meeting of the Italian Hospital was held on Saturday last, 27th ult., at the temporary hospital, 2, Orange Street, Red Lion Square, Cav. Dr. Castaneda presiding. As it is expected that the new premises now approaching completion will be opened, even if not complete in every detail, on March 14th next, the birthday of his Majesty the King of Italy, it is reasonable to hope that the meeting was held in temporary premises for the last time.

The new hospital is fully described in the report presented to the meeting. It is built on the site of two large freehold houses, given by the lamented founder, Commendatore John Ortelli, and is, appropriately, in the style of the Italian Renaissance. The façade looking into Queen Street is in red brick, divided by pilasters of white stone work, carved capitals supporting a heavily-moulded architrave dividing the public part of the building from the third floor, reserved to the nursing sisters. The mullions of the windows and the main entrance are also in stone. On the Devonshire Street side the circular main staircase, terminating in a beautiful chapel, surmounted by a dome and lantern, gives an admirable finish. The basement is fitted with the offices and conveniences necessary for such an institution. On the ground floor to the right is a fine board-room; to the left two rooms for the resident surgeon. Down the passage is a large waiting-room (for out-patients) with entrance from Devonshire Street, opposite which there is an up-to-date operating-room, well-lighted and aired, leading to the accident and surgical ward, in which there are two beds. At the end of the hall there is a room for the secretary, a large dispensary, and close to this again a room for the visiting physician. On the first floor the men's ward, looking on the square, holds fourteen beds, and has fine light and plenty of cross ventilation, with baths and the usual offices and conveniences entirely separated from the ward. In the Devonshire Street wing there is a ward for surgical cases of four beds, and four single wards for particular or special cases, together with a duty-room. Here, again, the offices are carefully detached. On the second floor, reserved to women, is a large ward with eight beds, and smaller wards for children, with a duty-room. On the Devonshire Street side of this floor there are two more wards, holding two beds each, and an isolation ward, should this become necessary. This ward, as well as the usual offices, is entirely cut off by an open passage, with a continuous and clear draught. On the third floor is the sisters' dormitory for eight beds, with separate rooms for dining, sitting, sewing, and small infirmary. On this floor, too, is the circular and domed chapel for their use, and that of the patients who may be convalescent. Here also is a covered way leading to a flat roof, where those well enough to ascend can get fresher air than even the wards themselves can give, and that recreation which may be helpful to their convalescence. All the floors are of concrete, and consequently fire-proof, the passages being paved with granolithic material and the floors of the several wards boarded in oak. The founder, in his intentions, spared no pains or money to complete this work of his heart and of his life, and though, unfortunately, he has passed away before seeing it terminated, his wife and executrix has taken care that her husband's wishes should be carried into effect. A passenger lift and electric lighting, unprovided for in the contract, as well as other incidental expenses are being defrayed by her personally.

During the rebuilding such part of the work as could be has been carried on at the premises in Orange Street, and the committee desired specially to record their thanks to the Homœopathic Hospital for help in bridging over the gap. The number of patients relieved during the year was 4,943, of whom 2,437 were English. The hospital makes no distinction either in nationality or religious faith. The ordinary income for the past year amounted to £1,512, of which £516 was the profit realised by the festival ball, £100 being a grant from the Prince of Wales's Fund toward furnishing the new building. The expenditure was £510, showing a credit balance for the year of £1,002. This was, of course, due to the fact that, owing to the re-building, there were no in-patients, and the money will come in most usefully for the various requirements of the new building. The report concludes with a warm recognition of the medical and nursing staffs, and the expression of hopes for the success of the new institution so soon to be launched.

The proceedings at the meeting were of the briefest character, the report and accounts being read and adopted without dissent, as were the customary votes of thanks, and a special one to Mrs. Ortelli.

